# *AIP* gene germline variants in adult Polish patients with apparently sporadic pituitary macroadenomas

**DOI:** 10.3389/fendo.2023.1098367

**Published:** 2023-02-10

**Authors:** Małgorzata Trofimiuk-Müldner, Bartosz Domagała, Grzegorz Sokołowski, Anna Skalniak, Alicja Hubalewska-Dydejczyk

**Affiliations:** ^1^ Chair and Department of Endocrinology, Jagiellonian University Medical College, Kraków, Poland; ^2^ Department of Endocrinology, Endocrine Oncology and Nuclear Medicine, University Hospital in Kraków, Kraków, Poland

**Keywords:** aryl hydrocarbon receptor-interacting protein, *AIP*, pituitary, adenoma, mutation

## Abstract

**Introduction:**

Up to 5% of all pituitary tumors are hereditary *e.g.* due to *MEN1* or aryl hydrocarbon receptor-interacting protein (*AIP*) genes mutations.

**Objectives:**

The study was aimed at the assessment of the frequency and characteristics of *AIP*-mutation related tumors in patients with apparently sporadic pituitary macroadenomas in the Polish population.

**Materials and methods:**

The study included 131 patients (57 males, 74 females; median age 42 years) diagnosed with pituitary macroadenomas, and with a negative family history of familial isolated pituitary adenoma (FIPA) or multiple endocrine neoplasia type 1 (MEN1) syndromes. Sanger sequencing was used for the assessment of *AIP* gene variants. The study was approved by the Ethics Board of JUMC.

**Results:**

*AIP* variants were identified in five of the 131 included subjects (3.8%): one diagnosed with Cushing’s disease, two with acromegaly, and two with non-secreting adenomas. Patients harboring hereditary *AIP* gene alterations did not differ from the rest of the study group in median age at diagnosis (41.0 vs. 42.5 years, P=0.8), median largest tumor diameter (25 vs. 24 mm, P=0.6), gender distribution (60.0% vs. 56.3% females, P=0.8), secreting tumor frequency (60.0% vs. 67.5%, P=0.7), or acromegaly diagnosis frequency (40.0% vs.37.3%, P=0.9).

**Conclusions:**

In our series of apparently sporadic pituitary macroadenomas, *AIP* gene variant carriers did not differ substantially from patients with negative genetic testing. A risk factor-centred approach to *AIP* genetic screening may result in missing germline variants. Considering the clinical impact of such genetic variants and their relatively low penetrance, it is, however, doubtful if general genetic screening benefits the whole cohort of pituitary macroadenoma patients and their families.

## Introduction

Pituitary tumors, if autopsy and radiological imaging are taken into consideration, occur in about 16.7% of the general population (1). The prevalence of clinically significant lesions is estimated at one case per approximately 1000 individuals (2, 3). Most of pituitary adenomas are sporadic (4). Only up to 5% of them are hereditary and can be caused, *e.g.* by *MEN1* or aryl hydrocarbon receptor-interacting protein (*AIP*) gene mutations, and present as a part of multiple endocrine neoplasia type 1 (MEN1) or familial isolated pituitary adenoma (FIPA) syndromes (4, 5).

The *AIP* gene is a suppressor gene encoding a 330 amino acid protein involved in the cAMP-phosphodiesterases pathway (6–11). The most common *AIP* variants (*AIP*var) are nonsense and missense mutations, deletions, insertions, splice-site and promoter mutations, and large deletions (6, 7). Most of them may result in a truncated protein or, less frequently, affect the tetratricopeptide repeat (TPR) domains or the C-terminal α-helix (6–8, 12, 13). Furthermore, in patients with germline *AIP*var, loss of heterozygosity (LOH) has been found in the tumor tissue at the site of the *AIP* gene in the 11q13 region (6, 8). Some of the *AIP*var are rare alterations without pathogenic effects, and no impact on protein function. Differentiating between these issues is important because rare genetic changes are also found in healthy controls (7). Whenever *AIP*var is used in this manuscript, it refers to pathogenic or possibly pathogenic variants of AIP.

About 90% of *AIP*var-related pituitary tumors are macroadenomas (14), mostly (80%) somatotropinomas and prolactinomas (6, 14, 15). Adenomas in patients carrying pathogenic *AIP*var are characterized by larger size, younger age at diagnosis (<30 years), aggressive growth, and resistance to treatment (4, 5, 14, 16). Low *AIP* protein expression is a better predictor of GH-secreting tumor aggressiveness than high Ki-67 index or p53 expression (4, 17, 18).

Our investigation was aimed at the assessment of the frequency and characteristics of pathogenic *AIP*var-related tumors in a population of Polish patients with apparently sporadic pituitary macroadenomas.

## Materials and methods

**Figure 1:**
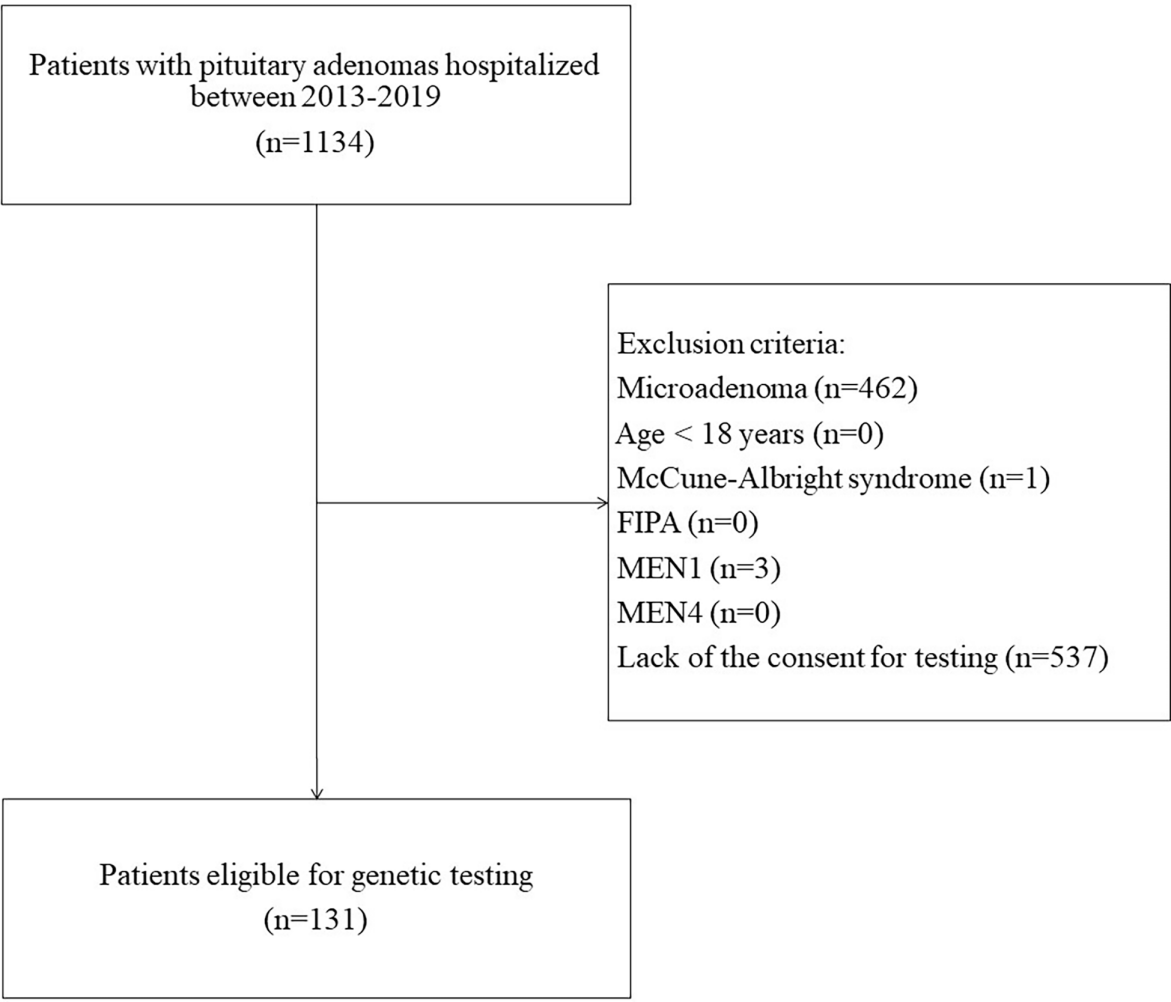
Flow chart of the patients’ inclusion.

This was a single-center study. Inclusion criteria were: (a) diagnosis of pituitary macroadenoma, (b) age >18 years, (c) negative family history of MEN1 syndrome, familial isolated pituitary adenomas (FIPA), and other hereditary syndromes with pituitary involvement, (d) informed consent to genetic testing. Between 2013 and 2019, 1134 patients with pituitary adenomas were hospitalized at the Department of Endocrinology specializing in adult care. Finally, after assessment for eligibility, 131 patients were included (**Figure 1**). Data on patients’ sex, age at diagnosis, tumor size, tumor type (clinical manifestation), pituitary surgery, and other treatments were recorded.

Written informed consent was obtained from all participants. The study design was approved by the Ethics Board of the Jagiellonian University (KBET/119/B/2013).

Genetic testing was performed between 2013 and 2019. DNA was isolated from peripheral blood samples. DNA sequencing was done using the Sanger method on an ABI3500 Genetic Analyzer. Analysis of the results was performed in SeqScape v2.7. All *AIP* coding exons and adjacent splice sites were analyzed. For variants, the clinical significance and prevalence in the population were determined based on publicly accessible databases (NCBI ClinVar, HGMD, VarSome), with NM_003977 used as a reference sequence (19–21).

Statistical analysis was performed with IBM SPSS Statistics 28.0. The Mann-Whitney U test was used for comparing the groups due to non-Gaussian distribution of data. *P* values <0.05 were considered statistically significant.

## Results

The study included 131 pituitary macroadenoma patients (57 males – 43.5%) diagnosed at 18-75 years (median age 42 years). Forty-two patients were diagnosed with GH-secreting tumors (32.1%), 21 (16.0%) with prolactinomas, 11 (8.4%) with ACTH-secreting tumors, 6 (4.6%) with gonadotropinomas, 1 (0.8%) with TSH-oma, and 7 (5.3%) with plurihormonal pituitary adenomas. The remaining 43 (32.8%) tumors were non-secreting.

### Patients without *AIP* gene germline variants

This group comprised 126 patients (96.2% of the study group), 55 males (43.7%). The median age at diagnosis was 42.5 years (range 18-75 years). The median largest tumor diameter in this group was 24 mm. 101 (80.2%) of *AIP*var-negative (*AIP*var(–)) patients underwent neurosurgical procedures (mostly transsphenoidal adenomectomy); the surgery was curative in 42 patients (41.6%). Among 47 patients with GH-secreting and plurihormonal tumors, 22 required prolonged treatment with somatostatin analogues (SSA).

### Patients with *AIP* gene germline variants

A germline *AIP*var was identified in five patients (3.8%): two with c.47G>A (p.Arg16His), two with c.911G>A (p.Arg304Gln) and one with c.684G>A (p.Gln228=). One patient was diagnosed with Cushing’s disease (9.1% of 11 ACTH-producing adenomas), two with acromegaly (4.8% of 42 GH-producing adenomas), and two with non-secreting adenomas (4.7% of 43 non-secreting tumors). There was no difference in secreting tumor (P=0.7), or acromegaly frequency (P=0.9) as compared to the group without pathogenic *AIP*var. This group consisted of two males and three females (no difference in gender distribution from the *AIP*var*(*–) group, P=0.8). The patients’ median age at diagnosis was 41 years (range 23-74 years) and did not differ from the *AIP*var*(*–) group (p=0.8). The median largest tumor diameter (25 mm) also did not differ significantly (P=0.6). Four out of 5 patients were treated surgically (80%), and the surgery was curative in one patient. One out of two patients diagnosed with acromegaly was treated with somatostatin analogues.

A brief description of the *AIP*var positive (*AIP*var(+)) patients and the predicted impact of the detected *AIPvar* are presented in **Table 1** and **Table 2**, respectively.

**Table 1:** Clinical description of *AIP*var(+) patients.

No of the patient	Gender; age at diagnosis	*AIP*var*; NCBI dbSNP accession number	Clinical description
I	Female; 74 years	c.47G>A (p.Arg16His) missense variant; rs145047094	**Symptoms:** proximal myopathy of the lower limbs, right eye ptosis, double vision, and signs of heart failure. **Imaging**: pituitary tumor of 25x18x22 mm in CT **Diagnosis:** Cushing’s disease. **Therapeutic approach first line:** transsphenoidal tumor resection **Histopathological examination:** corticotropic pituitary adenoma with Crooke cells (immunohistochemical staining: ACTH+, Ki67 index – approx. 3%) **Therapeutic approach second line:** stereotactic radiotherapy, ketoconazole for hypercortisolism (due to progressive tumor on MRI in the next hospitalization) **Follow-up:** the patient was lost for follow-up
II	Male; 41 years	c.47G>A (p.Arg16His) missense variant; rs145047094	**Symptoms:** phenotypical features of acromegaly **Imaging:** pituitary tumor of 10 x 14 mm in MRI **Diagnosis:** acromegaly **Therapeutic approach first line:** long-acting somatostatin analogue with subsequent transsphenoidal resection of the tumor **Histopathological examination**: pituitary somatotropic adenoma (immunohistochemical staining: GH+, PRL+/-, Ki-67 <1%) **Follow-up:** IGF-1 concentration was within the normal range, although there was no GH suppression in the oral glucose tolerance test. MRI showed no tumor recurrence.
III	Female; 26 years	c.911G>A (p.Arg304Gln) missense variant; rs104894190	**Symptoms:** amenorrhea, severe headaches, weakness, polydipsia, polyuria, galactorrhoea, and phenotypical features of acromegaly **Imaging:** pituitary tumor of approximately 3 cm in MRI **Diagnosis:** acromegaly **Therapeutic approach first line**: two pituitary surgeries (tumor size reduction) **Therapeutic approach second line**: radiotherapy and octreotide LAR. **Follow-up:** stable disease in the latest MRI (performed in 2018)
IV	Male; 56 years	c.911G>A (p.Arg304Gln) missense variant; rs104894190	**Symptoms:** an asthenia episode, right-sided ptosis, left-sided hemianopsia and periodic headaches; no abnormalities in laboratory tests. **Imaging:** pituitary lesion of 28x21x19 mm (compressing optic chiasm) in MRI. **Therapeutic approach first line:** pituitary surgery **Diagnosis:** non-functioning pituitary macroadenoma **Histopathological examination:** chromophobe adenoma (immunohistochemistry: positive staining for LH in some of the cells) **Therapeutic approach second line**: reoperation (residual tumor progression) **Therapeutic approach third line**: CyberKnife^®^ radiotherapy (residual mass of 25x19x17 mm in MRI) **Follow-up:** tumor residual mass at the end of follow-up (2018)
V	Female; 23 years	c.684G>A (p.Gln228=) synonymous variant; rs1365555914	**Symptoms:** headaches, visual field defects, weakness, lack of stamina, and mood deterioration; no abnormalities in hormonal check-up and ophthalmological examination **Imaging:** a 14x10x8 mm pituitary lesion in MR **Diagnosis:** non-functioning pituitary macroadenoma **Therapeutic approach first line:** active surveillance **Follow-up:** no tumor progression was observed during follow-up.

*All variants were identified in heterozygous state.

**Table 2:** Predicted impact of the detected *AIP* variants: disease-related classifications and *in silico* assessment of the impact on the AIP protein (according do prediction tools).

AIP variant	Classification according to ACMG criteria [25]*	ClinVar classification	HGMD	SIFT	Provean	MutationTaster	M-CAP	LRT	FATHMM	MVP	MetaSVM	MetaLR
c.47G>A (p.Arg16His) rs145047094	Benign	Conflicting interpretations of pathogenicity	associated with pituitary adenomas (CM071540)	Tolerated	Uncertain	Disease-causing	Damaging	Deleterious	Damaging	Pathogenic	Damaging	Damaging
c.911G>A (p.Arg304Gln) rs104894190	Benign	Conflicting interpretations of pathogenicity	associated with pituitary adenomas (CM070646)	Damaging	Neutral	Disease-causing	Damaging	Neutral	Tolerated	Uncertain	Tolerated	Tolerated

*as assessed by VarSome [21]. No data for c.684G>A (p.Gln228=) variant.

### Family screening

Two of the five *AIP*var(+) patients agreed for their families to be offered *AIP* genetic testing. The *AIP* alteration c.911G>A was found in the asymptomatic mother of patient III, who proved negative on hormonal check-ups and imaging. The second tested person was the underaged son of patient II. He carried an *AIP*var of unknown clinical significance, c.47G>A, and has not been clinically screened yet.

## Discussion

In this study, we wanted to investigate the frequency and characteristics of germline *AIP*var in Polish patients with apparently sporadic pituitary macroadenomas, followed up in the tertiary academic clinical center. We have found *AIP* variants that are very rare and/or have been described in the databases NCBI ClinVar or HGMD as pathogenic or of unknown significance in 3.8% of the studied group. Similar frequencies of germline *AIP*var were found in a study by Cazabat et al. They have found germline *AIP*var in 16 (3.6%) out of 443 (aged 4-87 years, both with micro- and macroadenomas) (22). The prevalence of *AIP*var(+) patients might be higher in younger (≤30 years) cohorts. In a study by Hernandez et al., 8.4% of young-onset sporadic pituitary adenoma cases harbored pathogenic or likely pathogenic *AIP*var (15). In a sporadic cohort of patients diagnosed with macroadenomas ≤30 years or with pituitary adenomas ≤18 years of age, Marques et al. found *AIP* alterations in 6.8% of cases (23). In contrast, in a Spanish cohort of 235 apparently sporadic adenomas in patients ≤30 years of age, pathogenic *AIP*var were detected in 3.8 of cases (24). The higher incidence of pathogenic mutations in some of the above-mentioned studies is mainly because the pediatric population was not included in our analysis. It should be noted that pathogenicity assessment may be based on different criteria, parameters, and tools. The missense variants identified in our study, designated as pathogenic or of unknown significance in the clinical databases NCBI ClinVar and HGMD, have been classified as (likely) benign according to ACMG 2015 criteria (25), as assessed by VarSome. The different assessment of pathogenicity may also be the cause of different variant frequency identification in the studies mentioned above.

Based on the collected data, we have found that our patients with *AIP*var did not differ substantially from the rest of the study group. In our study, the median age at diagnosis in *AIP*var(+) patients was 41.0 vs. 42.5 years in the rest of the group. This is inconsistent with most of the available data. In the series by Marques et al., patients with pathogenic *AIP*var were 8 years younger at the onset of symptoms and 6 years younger at diagnosis (23). 65% of them were younger than 19 years, and 87% were younger than 30 years. Daly et al. compared patients with or without *AIP*var and recorded a larger difference in age at diagnosis: 25.7 vs. 38.8 years, respectively (13). A similar age difference was found by Cazabat et al. (22): 23.5 vs. 40.9 years, respectively; none of the *AIP*var carriers was older than 40 years. This contrasts with our group, in which 3 out of 5 patients were older than 40 years. It may be argued that patients were younger at the onset of the disease, nevertheless, the oldest was over 70 years old when diagnosed with Cushing’s disease. Data may also be distorted by a large number of patients excluded due to lack of consent to participate in the study, exclusion of children and adolescents, and a delay in referring patients to the Endocrinology Department by other medical specialists due to the lack of specific symptoms.

We noted no difference in median tumor diameter, whereas, in published studies, the size difference is significant in favor of *AIP*var-related tumors, 24.6 ± 10.7 mm vs. 14.5 ± 10.1 mm in genetically unaltered cases (13). This may be because we only enrolled macroadenoma patients, and large tumors are predictive of germline *AIP*var (26). In the sporadic pituitary tumors cohort reported by Hernandez-Ramirez et al., there was no difference in the proportion of giant adenomas between *AIP*var positive and negative patients (15). All *AIP*var(+) sporadic cases had macroadenomas (in contrast to 86.3% in *AIP*var*(*–) group), and presented more frequently extrasellar extension (95% vs. 58.9%, respectively).

The available data indicates a small predominance of males (12, 16, 26) or an equal number of males and females (15, 27) among *AIP*var carries. In a large international collaborative study, *AIP*var carriers were predominantly males (63.5%) (16). Similar results were obtained in a smaller study of sporadic pituitary tumors (around 61% *AIP*var carriers being males) (12). Interestingly, male and female patients showed no phenotypic differences (12, 16). The gender proportions in our study were inverted: 3 *AIP*var(+) females to 2 males (similarly as in the whole screened group).

We have also noticed a deviation from other cohorts in the tumor types distribution. In our study, GH-secreting tumors were the most common (40% of *AIP*var(+) patients and 37% of the rest of the group). The data indicate that GH-secreting tumors appear more often in *AIP*var(+) cases, up to 78.1% of some studied cohorts (14, 16, 26). The proportion of *AIP*var(+) acromegalic patients was similar (4.8% vs. 4.1%) as reported by Cazabat et al., and higher in the case of ACTH-producing (9.1% vs. 6.8% in Cazabat’s study) or non-secreting/gonadotropin-secreting adenomas (4.7% vs. 0.9%, respectively) (22). We have not found any *AIP*var(+) patient with prolactinoma (in contrast to 4.6% of all prolactin secreting tumors in Cazabat study (22)). The group of Hernandez-Ramirez did not find any *AIP*var(+) patients with Cushing’s disease, functioning gonadotropinomas, or TSH-omas (15). In their cohort of sporadic *AIP*var(+) cases, all patients were diagnosed with acromegaly. In a sporadic cohort of young patients, *AIP*var-related tumors accounted for 10.5% of somatotropinomas, 1.3% of prolactinomas, and none of the non-functioning adenomas (23).

Our group of *AIP*var(+) patients differs from those reported in the literature in the clinical presentation (**Table 1**) and age at diagnosis. This may be explained by the small group size and selection bias, particularly screening-out patients diagnosed during childhood, who comprise a large proportion of the other cohorts (22). Most of the patients who agreed to participate were referred for hospital workup, which may explain the underrepresentation of prolactinomas.

Surgery and SSA treatment are less effective in *AIP*var(+) cases (5, 16). Leontiou et al. evaluated the response to SSA in AIPvar(+) patients and found that 53% had a poor response to therapy (27). In contrast, resistance to SSA therapy is noted in about 25% of AIPvar (–) patients (27–29). In another study, more than one-third of patients with *AIP*var-related somatotropinomas underwent two or more surgical interventions. Furthermore, only 11% achieved disease control during post-operative SSA treatment; tumor shrinkage was observed in 16% of cases (5). In Marqes et al. study *AIP*var-related adenomas more frequently required multimodal and multiple treatments (23). Similar tumor behavior was noticed in our group. Only one patient was followed without any interventions so far. Most of the presented cases, like in other reports (4, 5, 14, 16), required a multimodal therapeutic approach, which frequently did not result in proper disease control. The considered reasons for treatment resistance in *AIP*var(+) tumors are defective Gai2 or ZAC1 pathways, mediating SST2 receptors function (18, 30). It is likely that decreased *AIP* expression is the cause of the poor response of GH-releasing tumors to SSA treatment (18, 30, 31).

In our study, we also wanted to assess the impact of the three *AIP*var types detected in our group, on the course of the disease and compare the results with the available literature.

Patients I and II harbored the c.47G>A (p.Arg16His) missense *AIP*var (rs145047094). In the non-Finnish European population, the variant occurs with a frequency of 0.34%, according to the gnomAD v2.1.1 database (32). The pathogenicity interpretation according to ACMG guidelines (25) (assessed by VarSome (21)), ClinVar and HGMD databases, as well as *in silico* classifications of the variant are summarized in **Table 2**.

The summary of published data on the clinical significance of the c.47G>A variant is presented in **Table 3**. Most authors have interpreted this alteration as a very rare variant without a clear causative effect (**Table 3**). Interestingly, this variant was noted in patients with Cushing’s disease from Poland, as well as in a young patient with Cushing’s disease in Cazabat et al. cohort (22, 33). Similarly, patient I in our group was diagnosed with Cushing’s disease due to an aggressive type of adenoma (Crooke cells in IHC staining: ACTH+, Ki67 about 3%), refractory to treatment. On the other hand, in patient II with acromegaly, the adenoma showed no signs of increased aggressiveness on histopathological examination; however, he did not fulfil all acromegaly remission criteria. This variant was also found in the patient’s son, but he is yet to be evaluated clinically. Regarding offspring age, only longitudinal observation may prove if the variant will be pathogenic. Therefore, the impact of the c.47G>A variant on pituitary tumorigenesis still needs to be elucidated.

**Table 3:** Published data on clinical significance of *AIP* c.47G>A variant.

First author and year of publication	Study description	Description of clinically affected carriers	Description of non-affected carriers (if available)	Authors’ verdict on clinical significance of variant
Daly et al. (2007) (13)	MC; 73 FIPA families, 156 patients with PA.	Two first cousins with acromegaly (MiA); age at diagnosis known for one carrier (46 years).		First description of the variant Impact on the structural and functional status of AIP protein needs to be determined. Variant at the time of the study not described in non-FIPA individuals screened for *AIP* polymorphisms.
Georgitsi et al. (2007) (33)	MC; 460 PA patients and patients from families with MEN1 features (including 122 unselected PA patients from Poland). Control groups: 90 German, 288 UK Caucasian, 110 Caucasian (Centre d’Etude du Polymorphism Humain, The Netherlands), 52 Italian and 209 Finnish healthy subjects.	1/71 Italian acromegaly patients (no LOH in tumor tissue). 1 unselected PA patient (of Polish descent) from United States (no LOH in tumor DNA). 3/122 Polish PA patients (all diagnosed with Cushing disease)	1/90 German controls	A neutral variant
Cazabat et al. (2007) (34)	SC; 154 sporadic acromegalic patients. Control group: 270 subjects	2 acromegalic patients	Variant found in 2 controls	Rare variant (although authors cannot exclude its pathogenic impact)
Buchbinder et al. (2008) (35)	SC; 110 patients with sporadic PA	M, 55 years at diagnosis, NF MA. F, 48 years at diagnosis, NF MiA.	Family screening available for the second patient: 28 years old son negative on MR screening; father – putative variant carrier – died of lung cancer.	Evaluation of oncogenic impact not possible
Guaraldi et al. (2011) (36)	SC; Case report	F, recurrent acromegaly, 37 years at diagnosis (TD 10 mm). Acromegaly in 4 paternal relatives; 2 available for genetic testing – AIP c.47G>A variant was not detected. This change was also not present in the patient’s mother.		More likely rare variant
Tichomirowa et al. (2011) (5)	MC; 163 sporadic macroadenoma patients diagnosed at the age <30 years.	F, acromegaly, 29 years at diagnosis. M, prolactinoma, 20 years at diagnosis, TD 45 mm.		Most likely a rare variant (based on literature review).
Cazabat et al. (2012) (22)	SC; 443 sporadic PA patients. Control group: 360 French subjects.	M, Cushing disease, 14 years at diagnosis; MiA.		Variant not found in control group. Authors considered the variant probably pathogenic (due to previous reports and young age at diagnosis of the patient).
Baciu et al. (2013) (37)	SC; case report. Control group: 108 subjects without clinical evidence of pituitary disease.	M, 38 years at diagnosis, NF MA, TD 38 mm; additionally diagnosed with intellectual disability.	Family: 1 sister tested genetically – negative; family history negative for PA. Control group: variant found in a F, 20 years of age, with follicular thyroid neoplasm on FNAB.	Benign role of the variant cannot be conclusively demonstrated – defined as variant of unknown significance.
Zatelli et al. (2013) (38)	SC, large Italian FIPA family, genetic testing in 16 members of the family. Control group: 16 sporadic acromegalic patients and 6 subjects without PA.	F (proband), 22 years old at diagnosis, NF MiA (TD 5 mm). F, 36 years old at diagnosis, NF MiA (TD 2 mm), maternal aunt of the proband.	6 carriers negative at clinical and MRI screening, aged 73, 44, 46, 18, 9, and 6 years (including the proband’s mother).	A rare variant. Comment: paternal cousin of the proband died at the age of 32 years of aggressive NF PA.
Preda et al. (2014) (39)	SC; 127 sporadic patients with PA diagnosed <40 years	F, acromegaly, MA with aggressive features (increased MIB1, sparse granulation pattern), 29 years at diagnosis		Pathogenicity remains unclear (literature association with aggressive NF adenoma).
Ferraù et al. (2015) (40)	MC; 215 acromegalic patients (including 5 FIPA cases).	F, 26 years at diagnosis; MiA; FIPA – previously described by (31); although there is a discrepancy in the patient’s age at diagnosis and information on secretory activity of the tumor. M, 50 years at diagnosis, MA.		No conclusion on *AIP* c.47G>A significance given by the authors.
Hernandez-Ramirez et al. (2015) (15)	MC; 1725 individuals (1231 from FIPA cohort and 494 from sporadic cohort), 906 patients affected with PA (502 from familial and 404 from sporadic cohort)	2 from sporadic cohort.		Not pathogenic.
Araujo et al. (2017) (41)	SC; 132 sporadic PA (MA <40 years of age; PA of any size < 18 years).	M, acromegaly, 33 years at diagnosis, TD 12 mm.		Rare non-pathogenic variant (rare polymorphism), as no LOH was found in tumor samples (literature data).

F, female; FIPA, familial isolated pituitary adenomas; M, male; MA, macroadenoma; MC, multicenter study; MiA, microadenoma; MR, magnetic resonance; NF, non-functioning; PA, pituitary adenoma; SC, single center study; TD, tumor diameter.

While examining the mechanisms of the pathogenicity of the rs145047094 variant, Baciu et al. concluded that pathogenic variants which lead to premature stop codons, cause truncating AIP proteins and affect important functional domains; however, missense changes can have both pathological and mild effects (37). Pituitary tumorigenesis may require other pathology, for example, the LOH in adenoma cells, which was not observed in the case of the above-mentioned variant (13, 33–35). It has also been suggested that this variant lacks functional impact due to the weak binding effect of PDE4A5 (6). In a latest work of Garcia-Rendueles et al., who performed a series of functional *in vitro* analyses, N-term and C-term *AIP* point variants were proven to impact the molecular interactions of *AIP* and block the RET-apoptotic pathway. Based on those criteria, the variant p.Arg16His was classified as pathogenic (42).

Another missense variant, c.911G>A (p.Arg304Gln), was detected in our study in a 26-year-old acromegalic woman and a 56-year-old man with non-secreting adenoma (patients III and IV). It is registered in NCBI dbSNP under the accession number rs104894190. For pathogenicity classification and *in silico* analyses, see **Table 2**. This variant occurs at a CpG island hotspot (5–7). The clinical significance of the c.911G>A variant is equivocal (**Table 4**).

**Table 4:** Published data on clinical significance of *AIP* c.911G>A variant.

First author and year of publication	Study description	Description of clinically affected carriers	Description of non-affected carriers (if available)	Authors’ verdict on clinical significance of variant (if available)
Georgitsi et al. (2007) (33)	MC; 460 PA patients and patients from families with MEN1 features (including 122 unselected PA patients from Poland). Control groups: 90 German; 288 UK Caucasian; 110 Caucasian (Centre d’Etude du Polymorphism Humain, The Netherlands); 52 Italian and 209 Finnish healthy subjects.	1/122 Polish PA patients; Cushing’s disease; 26 years at diagnosis; gender and TD not specified. (Authors have noted that they identified the same variant in an Italian patient with acromegaly – unpublished data.)		First description of the variant. Pathogenic (also because not identified in the control group).
Cazabat et al. (2007) (34)	SC; 154 sporadic acromegalic patients. Control group: 270 subjects.	F, acromegaly, 37 years at diagnosis (first manifestation – pituitary apoplexy).		Variant not detected in controls; functional consequences should be evaluated.
Leontiou et al. (2008) (27)	MC; 67 FIPA patients (26 families) and 85 sporadic PA patients. Control group: 96 European and 78 Japanese subjects.	2 F FIPA relatives from Romania (acromegaly); aged 30 and 17 at diagnosis.		
Igreja et al. (2010) (6)	MC; 64 FIPA families (26 previously described (19)).	Additionally to (19): F, NF MA, 52 years at diagnosis. F, prolactinoma, MA, 23 years at diagnosis. (samples from other family members not available)		Pathogenic
Occhi et al. (2010) (43)	MC; 131 sporadic acromegalic patients and probands of 6 FIPA families. Control group: 250 healthy subjects.	F, acromegaly, 67 years at diagnosis, MA (TD 23 mm), multiple other tumors. F, acromegaly, 38 years at diagnosis, MiA (TD 3 mm).		Variant not detected in the healthy controls. Variant of reduced pathogenicity.
Tichomirowa et al. (2011) (5)	MC; 163 sporadic macroadenoma patients diagnosed at the age <30 years.	M, prolactinoma, 15 years at diagnosis, MA (TD 27 mm).	7 mutation carriers in family, no PA.	Divergent data from clinical and experimental studies (only modest effect being seen on functional assays).
Cazabat et al. (2012) (22)	SC; 443 sporadic PA patients; Control group: 360 French subjects.	F; prolactinoma (25 years at diagnosis); MiA		Considering benign clinical presentation and the frequency of microprolactinomas, the presence of the variant may be a coincidence
Cuny et al. (2013) (44)	MC; 174 patients with sporadic MA, diagnosed <30 years of age and without hypercalcemia.	M, prolactinoma, 27 years at diagnosis, TD 35 mm.		Pathogenic, despite the low score in *in silico* predictions.
Freudenberg-Hua et al. (2014) (45)	SC; 44 centenarians.		Variant detected in subjects without apparent pituitary tumor	
Schöfl et al. (2014) (46)	MC; 197 acromegalic patients diagnosed ≤30 years of age (91 tested for *AIP*var).	M, 29 years at diagnosis, positive family history, invasive MA.		Pathogenic.
Preda et al. (2014) (39)	SC; 127 sporadic patients with PA diagnosed <40 years.	M, acromegaly, MA with aggressive features (dense granulation pattern), 41 years at diagnosis; symptoms dating from 35 years of age. Proband’s younger sister diagnosed at screening with macroprolactinoma.	Proband’s older sister and 14 years old daughter (asymptomatic at testing).	Pathogenicity remains unclear.
Ferraù et al. (2015) (40)	MC; 215 acromegalic patients (including 5 FIPA cases).	F, 53 years at diagnosis, MiA. F, 62 years at diagnosis, MA. F, 67 years at diagnosis, MA (previously described (35)).		Conflicting evidence.
Hernandez-Ramirez et al. (2015) (15)	MC; 1725 individuals (1231 from FIPA cohort and 494 from sporadic cohort); 906 patients affected with PA (502 from familial and 404 from sporadic cohort).	23 patients, 20 from familial and 3 from sporadic cohort.		Pathogenic.
Araujo et al. (2017) (41)	SC; 132 sporadic PA (MA <40 years of age; PA of any size < 18 years).	M, prolactinoma, 18 years at diagnosis, TD 60 mm (LOH analysis was not performed).	Father and paternal uncle, negative on clinical, hormonal, and MR screening.	Likely pathogenic or variant of unknown significance
Tuncer et al. (2018) (47)	SC; 97 sporadic PA, symptoms onset ≤40 years of age.	M, acromegaly, 45 years at diagnosis, 38 years at symptoms onset, MA (densely granulated pattern, TD 50 mm). F, prolactinoma, 22 years at diagnosis, 16 years at symptoms onset, invasive MA (TD 22 mm; Ki67- 4%).		Pathogenicity uncertain (based on literature).
Dal et al. (2020) (48)	SC; one FIPA family (159 individuals – five generations; genetic screening in 72 surviving family members).	M, acromegaly, 37 years at diagnosis, no distinct PA on MR (probable pituitary apoplexy 10 years before diagnosis), subsequent MR revealed MiA. F, acromegaly, 37 years at diagnosis, MA (TD 10 mm), paternal aunt of the first patient.	50 carriers, the oldest 80-year-old male. Some of the family members exhibited acromegalic traits regardless *AIP* status. One carrier diagnosed during follow-up with NF intrasellar cystic lesions (TD 7 mm).	Disease penetrance 6%.

F, female; FIPA, familial isolated pituitary adenomas; M, male; MA, macroadenoma; MC, multicenter study; MiA, microadenoma; MR, magnetic resonance; NF, non-functioning; PA, pituitary adenoma; SC, single center study; TD, tumor diameter.

Dal et al. have postulated the role of other genes in the development of pituitary tumors in *AIP*var carriers, namely *PDE11A* (associated with adrenal tumorigenesis) and *ALG* (coding a protein essential for glycoprotein folding and stability) (48). Patient III from our study had a treatment-resistant tumor and required two surgeries, radiotherapy, and SSA treatment. The mother of the patient had the c.911G>A (p.Arg304Gln) variant but she was negative on biochemical check-up and imaging, which may question the variant’s clinical pathogenicity. Patient IV required double surgery and stereotactic radiotherapy, which may suggest that the variant impacts the clinical course of pituitary adenomas. While examining the mechanism of the mutation some authors found that this variant did not significantly reduce PDE4A5 binding (6), other concluded that it may impact AIP and aryl hydrocarbon receptor (AHR) interactions (33) or may interfere with protein stability or folding (49) without directly affecting the protein-protein interaction (6). Other authors observed that this *AIP* alteration did not disrupt chaperone binding and did not show a significant reduction in β-galactosidase activity, which may reduce its pathogenic effect (6, 50). Hernández-Ramírez et al. also concluded that the pathogenicity of the c.911G>A variant is uncertain (51). Aflorei et al. in their *in vivo* tests using *Drosophila melanogaster* models, decided that both the p.Arg16His and p.Arg304Gln variants should be assessed as non-pathogenic (52). Dal et al. estimated the penetrance of c.911G>A variant at 6% (48). In the study by Garcia-Rendueles et al., the functional outcome in transfected cells was similar as in the case of the variant p.Arg16His, therefore, the disease-causing potential of the variant p.Arg304Gln was also proven positive in this study (42).

The last variant was c.684G>A (rs1365555914), which was found in patient V. The pituitary tumor in this case was smaller than in the previous patients (I-IV), and subsequent pituitary MRIs did not reveal its progression over the years. The c.684G>A variant itself does not introduce an amino acid change (CAG>CAA, Lys>Lys), however, due to the additional common variant rs641081 (c.682C>A) in homozygous state in the patient, the final codon at position 228 in the protein in this patient changed from CAG to AAA, leading to the amino acid alteration p.Gln228Lys. The p.Gln228Lys alteration caused by the common variant rs641081 alone has been classified as benign, and the more prevalent allele A (observed in a homozygous state in the patient) occurs in the non-Finnish European population with a frequency of 99.8%. The variant rs1365555914 detected in the patient is extremely rare, with no information on its frequency available and zero allele counts reported in the gnomAD database for any population. This rare variant does not change the coded amino acid with any of the alleles of the rs641081 variant and, although there is a noticeable difference in the codon usage frequency for the reference glutamine (35.5 vs 14.1 per thousand for CAG and CAA, respectively), the usage frequency for lysine codons with the rs1365555914 variant, as observed in the patient, are very similar: 31.8 vs 27.5 per thousand for AAG and AAA, respectively (53). It is, therefore, not clear whether or not this variant is of clinical significance for the patient. However, we report it due to its rarity and because it was the only suspected variant identified in the genetic screening of the patient diagnosed at a young age.

The above-discussed *AIP* alterations illustrate the difficulties in genetic testing results interpretation. The inconsistency in assessing the pathogenic impact of *AIP* variants is particularly seen in simplex cases (54). Negative family history may be caused by reduced penetration of the variant or lack of information about the family (55). The penetration of pituitary adenoma in *AIP*var carriers is described in the range 12-30% (14, 55). Some studies even indicate that the pathogenic variant type has little effect on penetration (15, 55).

The question remains, what significance for clinical management each type of *AIP*var has. Daly et al. asked whether dividing *AIP* variants into non-pathogenic or pathogenic is useful (56). It is unclear whether *AIP* alterations are always the main factor responsible for tumor development. It may be that *AIP* variants only facilitate tumor formation. Variants considered clinically pathogenic may be reclassified as innocent on further analysis (56). Therefore, rare alterations deserve special attention. In our study, regardless of the specific type of the variant and its pathogenicity described in literature, most of the *AIP*var-related tumors were aggressive and usually resistant to standard treatment. Other data suggest that the type of changes in the *AIP*-encoded protein caused by mutation may be relevant for disease course and the decision on family member screening. According to Hernandez-Ramirez et al., truncating mutations are related to a younger age at diagnosis and the onset of symptoms, and a more common occurrence of pediatric cases (15). In this study, there was no difference between truncating and nontruncating mutations in the proportion of acromegaly, the number of patients per family, maximum tumor diameter, or extrasellar expansion.

During the genetic screening, new variants/mutations of the *AIP* gene may be found, and their pathogenicity usually needs to be proven. For example, in the study by Cazabat et al. (22), previously not described variants were found in half of sporadic AIPvar(+) patients. The authors performed parental screening in 7 out of 16 *AIP*var(+) patients. In all of them, one of the parents was an asymptomatic carrier. Of note, no pituitary adenoma was found in parents-carriers, who agreed to clinical evaluation (22). Ten new, likely pathogenic mutations were also reported by Hernandez-Ramirez’ group (15).

The important question is the probability of pituitary adenoma development in asymptomatic *AIP*var carriers. In a group of 160 apparently unaffected *AIP*var carriers in the Hernandez-Ramirez study, pituitary adenoma was established in 11.3% of patients (15). Half of them exhibited GH oversecretion, the rest were diagnosed with non-functioning adenomas. Only five out of 18 prospective cases were diagnosed with macroadenomas. The authors concluded that depending on the applied clinical screening, up to 25% of apparently unaffected carriers may develop pituitary adenoma. Marques et al. prospectively followed 187 apparently unaffected *AIP*var carriers identified by testing of the first-degree relatives of *AIP*var(+) FIPA and sporadic adenoma patients (23). 88.2% of them did not develop a pituitary adenoma during the mean 5.9 ± 3.3 years follow-up. 19 of 22 adenomas were diagnosed at first screening (in 8 cases, retrospective signs which may be attributed to the pituitary tumor were noted), and 3 cases were recognized during subsequent follow-up. The prospectively recognized pituitary adenomas were smaller, 68% being microadenomas, and were associated with lower rates of hypopituitarism at diagnosis, extrasellar extension, or cavernous sinus invasion. Such patients were less frequently operated and none of them required radiotherapy.

Finally, it is worth asking which patients should be screened, considering the varying course of the disease and low disease penetration in patients with *AIP*var. Genetic screening was most commonly indicated in the case of (12, 14, 26): meeting the criteria of FIPA, or pituitary adenoma diagnosed in <18 year-olds, or pituitary macroadenoma diagnosed in <30 year-olds. The probability of detecting new *AIP* alterations in the fifth decade of life is low (2, 12, 15). Published data suggest that only 13.2% of *AIP*var(+) patients had an onset of illness after 30 years of age (15). Therefore, unselected screening is probably not a cost-effective method (22, 27, 57–59). In large unselected cohorts, *AIP* pathogenic or likely-pathogenic variants occurred in 3.6%-8.3% of included patients (22, 27, 57, 59, 60). If young populations were considered, the incidence increased from 11.7% in patients <30 years to 20.5% in pediatrics (5). Although in our group, *AIP* variants were detected in older patients with an aggressive course of the disease, the benefit for the individual patient over 40-50 years of age from genetic screening is negligible if the family history is negative. It seems that the detection of an *AIP*var in this setting does not impact the patients’ management, as the *AIP*-encoded protein is not a therapeutic target currently. Even if a more aggressive course of adenomas in *AIP* germline variant-carriers may be predicted, the treatment modalities did not differ from those applied in large to giant, invading or drug-resistant pituitary tumors unrelated to *AIP* alterations.

In a systematic review by van den Broek et al. (26), the following recommendations were proposed based on available studies. The authors strongly recommend against routine genetic testing in sporadic pituitary adenoma. A weak recommendation for *AIP* mutation analysis in patients with sporadic pituitary adenomas 30 years old or younger, especially those diagnosed with acromegaly and gigantism, was made based on low-quality evidence.

## Conclusion

In conclusion, in our series of apparently sporadic pituitary macroadenomas, *AIP*var carriers were identified in 3.8% of the study group and did not differ substantially from patients with negative genetic testing. Therefore, routine genetic screening for *AIP* variants in non-selected adult pituitary adenoma patients seems currently ineffective. It seems that in clinical practice, a targeted screening approach limited to patients at risk of AIP-related pituitary adenomas should be applied. If an *AIP* variant is detected, genetic testing should be discussed with the proband’s family members (particularly symptomatic and younger ones), as additional data may improve our understanding of the clinical significance of detected genetic alteration.

## Data availability statement

The raw data supporting the conclusions of this article will be made available by the authors, without undue reservation.

## Author contributions

Study design: MT-M. Data collection: MT-M, GS. Data analysis: MT-M, BD, AS. Manuscript drafting: MT-M, BD, AS. Manuscript revision: MT-M, BD, GS, AS, AH-D. All authors contributed to the article and approved the submitted version.
